# Joint Timekeeping of Navigation Satellite Constellation with Inter-Satellite Links

**DOI:** 10.3390/s20030670

**Published:** 2020-01-25

**Authors:** Leyuan Sun, Wende Huang, Shuaihe Gao, Wei Li, Xiye Guo, Jun Yang

**Affiliations:** 1 College of Intelligence Science and Technology, National University of Defense Technology, Changsha 410073, China; sly_nudt@163.com (L.S.); 15574944081@163.com (W.H.); HJiao0513@163.com (X.G.); 2 State Key Laboratory of Geo-Information Engineering, Xi’an 710000, China; 3 Southwest China Research Institute of Electronic Equipment, China Electronics Technology Group Corporation, Chengdu 610000, China; JJ_paper@yeah.net; 4 National Time Service Center, Chinese Academy of Science, Xi’an 610000, China; lipinggg0803@163.com

**Keywords:** joint timekeeping, inter-satellite link time, timescale algorithm, frequency stability, predictability

## Abstract

As a system of ranging and positioning based on time transfer, the timekeeping ability of a navigation satellite constellation is a key factor for accurate positioning and timing services. As the timekeeping performances depend on the frequency stability and predictability of satellite clocks, we propose a method to establish a more stable and predictable space time reference, i.e., inter-satellite link time (ISLT), uniting the satellite clocks through inter-satellite links (ISLs). The joint timekeeping framework is introduced first. Based on the weighted average timescale algorithm, the optimal weights that minimize the increment of the ISLT timescale are determined and allocated to the clock ensemble to improve the frequency stability and predictability in both the long and short term. The time deviations with respect to the system time of nine BeiDou-3 satellites through multi-satellite precise orbit determination (MPOD) are used for joint timekeeping evaluation. According to the Allan deviation, the frequency of the ISLT is more stable than the nine satellite clocks in the short term (averaging time smaller than 7000 s), and its daily stability can reach 6 × 10^−15^. Meanwhile, the short-term (two hours) and long-term (10 h) prediction accuracy of the ISLT is 0.18 and 1.05 ns, respectively, also better than each satellite clock. Furthermore, the joint timekeeping is verified to be robust against single-satellite malfunction.

## 1. Introduction

Developed for several decades, global navigation satellite systems (GNSSs), including the US Global Positioning System (GPS), the Russian Globalnaya Navigazionnaya Sputnikovaya Sistema (GLONASS), the European Union Galileo system, and the Chinese BeiDou navigation system (BDS), have been established [1,2]. As a system of ranging and positioning based on time transfer, each GNSS has established and kept its own system time, e.g., the GPS system time (GPST) and the BeiDou system time (BDT). Besides the ground facility clocks, the spaceborne clocks on board of the navigation satellites are synchronized to their respective system times. That is, all the BeiDou satellite clocks are traced to the BDT by a satellite–ground time comparison and clock deviation prediction. For on-ground navigation users, the timekeeping ability of the navigation satellite clock is a key factor for accurate positioning and timing services. The timekeeping performance is limited by the frequency stability and predictability of the clock. In the present operation mode, the local time is separately kept by each satellite [3,4].

With the ultimate purpose of autonomous operation, the new generation of GNSS has been equipped or is planning to be equipped with inter-satellite links (ISLs) that implement inter-satellite ranging and communication [5]. To mitigate the spatial limitations of regional monitor networks, BeiDou ISLs have been used for time transfer from stations to overseas satellites. Inter-satellite time transfer is implemented with the ISL system of dual one-way ranging. The parameters of orbits and clock deviations are successfully decoupled and estimated separately [6,7]. According to the analysis of [8], the precision of BeiDou-3e inter-satellite clock deviations is superior to 0.3 ns and coincidental with satellite–ground clock deviations when channel delays are corrected.

In this research, we propose a more comprehensive method of joint timekeeping by using a navigation satellite clock ensemble. The inter-satellite time deviation is used to establish a more stable and predictable synthetic timescale by using a timescale algorithm. The synthetic timescale based on ISLs is defined as the inter-satellite link time (ISLT). The most common timescale algorithms are the weighted average and Kalman filtering algorithms, from which some algorithms are modified. The classical weighted average timescale algorithms include ALGOS, which is used by the International Bureau of Weights and Measures (BIPM) [9], and AT1, which is used by the National Institute of Standards and Technology (NIST) [10], both of which include goals of setting up basic timescale equations. With the input of time deviations, the weighted average algorithm optimizes the frequency stability of the synthetic timescale by adjusting the weights of individual clocks. Kalman filtering algorithms began to be applied to timescales in the 1970s. By the early 1980s atomic time TA(NIST), one of the most broadly used early timescales, had been established by using these algorithms [11,12]. The Kalman timescale is mainly determined by a physical clock with the most stable frequency in the long term and ignores frequency stability in the short term. For the comprehensive utilization of frequency stability in both long-term and short-term of atomic clocks, Weiss [13] proposed the AT2 timescale algorithm, which combines the AT1 algorithm and Kalman filtering. The AT2 algorithm refactored time deviations with frequency deviations, which were estimated by a Kalman filter, and then formed a synthetic timescale by weighted averaging. The Kalman plus weights (KPW) algorithm of Greenhall [14] assumed that there was only white frequency noise (WFM) left in the time deviation that was corrected by the Kalman frequency, and the weights were adjusted according to the intensity of WFM. For a similar purpose, Greenhall [15] presented a reduced Kalman timescale algorithm and proved that the algorithm could minimize the variance of the timescale increment in theory.

In the following sections, a joint timekeeping framework with satellite clocks is introduced, followed by an ISLT timescale algorithm. The resulting improvements of the frequency stability and predictability of the ISLT over the satellite physical atomic clocks are then highlighted.

## 2. Joint Timekeeping Framework

The GNSS system time reference is established and kept by atomic clocks in ground operation control systems or are joined by the satellite clocks. The local time of each satellite is maintained by its spaceborne clock, and it is synchronized to the GNSS system time by two-way satellite time and frequency transfer (TWSTFT) or by multi-satellite precise orbit determination (MPOD) [3,16,17], which is called distributed timekeeping. The prediction parameters of clock deviations are broadcast in the ephemeris for positioning and timing. In this section, the establishment method of the inter-satellite link time is introduced and the improvement is evaluated in brief compared to the distributed timekeeping.

### 2.1. Traceability by Prediction

The ISLT unites the satellite clocks and is established through inter-satellite time transfer and communication. All the navigation satellite clocks are traced to the ISLT, especially in the mode of autonomous operation (i.e., operation without support of ground stations). As the ISLT is broadcast, it should confirm synchronization to the GNSS system time, which is steered to the local realization of the Coordinated Universal Time (UTC), modulo 1 s, according to the recommendations of the International Telecommunication Union (ITU) and the General Conference on Weights and Measures (CGPM) [18]. When there is no real-time satellite–ground time transfer link, the synchronization between the ISLT and GNSS system time should be realized by a prediction of the timescale deviation. The satellite clocks, of course, can be predicted and synchronized to the GNSS system time. However, navigation performance will degrade as the divergence of prediction errors increases due to the unstable frequency of spaceborne clocks. The objective of this work is to form a more stable and predictable synthetic clock as the accurate time reference of the navigation constellation, i.e., ISLT, to restrain the degradation of navigation performances.

### 2.2. Establishment of ISLT

**Figure 1:**
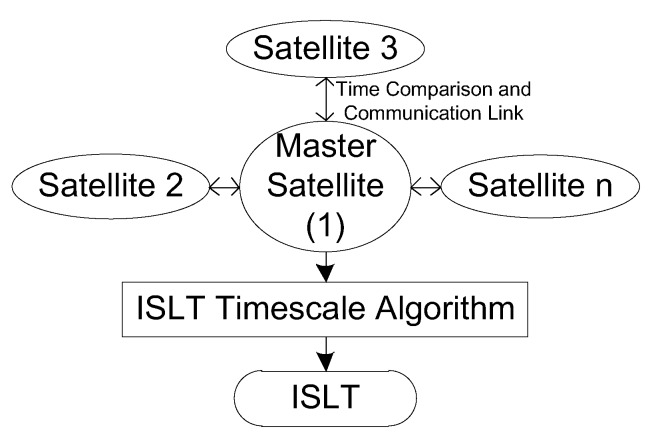
A brief establishment framework of inter-satellite link time (ISLT).

The ISLT is formed as a synthetic clock by utilizing the frequency stability of an ensemble of satellite clocks. One satellite is selected as the master satellite, whose working clock is noted as the reference clock, and other satellites are named as slave satellites. It should be stated that the reference clock does not mean the time reference of the constellation; rather, it is a center node of time comparison. All the slave satellites compare their clocks with the reference clock through ISLs periodically. The time comparison is limited by inter-satellite visibility. The satellite visible to the master satellite is defined as an anchor satellite that is able to directly compare time through an ISL. The one not visible to the master satellite must compare time by the relay of an anchor satellite. The time comparison results are collected by the master satellite and used to generate the ISLT with a timescale algorithm. A brief establishment framework of the ISLT is shown in Figure 1. *n* is the number of satellites that participate in the joint timekeeping, and the master satellite is numbered 1.

As the clock reading is unmeasurable, the ISLT is represented as the time deviation relative to the reference clock. With the support of ground operation control systems, the ISLT operates in parallel with and is synchronized to the GNSS system time. The time deviation of the ISLT relative to the GNSS system time is calculated by:

(1) $$x_{e}=x_{e1}+x_{1}$$

where $x_{e1}$ is the time deviation of the ISLT generated by the ISLT timescale algorithm relative to the reference clock and $x_{1}$ is the time deviation of the reference clock relative to the GNSS system time, which is estimated through a time comparison link between the master satellite and the ground station. The subscript “*e*” stands for the synthetic clock (ISLT). As different types of time deviations are involved in the following parts, the deviation relative to the GNSS system time is especially noted as the absolute time deviation.

In keeping with the actual navigation application, a prediction model of the ISLT is estimated with its historical absolute time deviation data and used for prediction. Combining the real-time results of the ISLT timescale algorithm, the absolute time deviation of the reference clock is predicted as:

(2) $${\hat{x}}_{1}={\hat{x}}_{e}-x_{e1}$$

where ${\hat{x}}_{e}$ is the predicted value of the time deviation of the ISLT. The absolute time deviation of each slave satellite is estimated based on the inter-satellite time comparison:

(3) $${\hat{x}}_{i}={\hat{x}}_{1}+x_{i1},\;i=2,3,\ldots ,n$$

where $x_{i1}=x_{i}-x_{1}$ is the time deviation of slave satellite *i* relative to the master satellite obtained by an inter-satellite time comparison. It is obvious that the indirect prediction is more accurate than direct prediction of satellite clocks, when the ISLT is more stable and predictable than the satellite clock.

## 3. ISLT Timescale Algorithm

The function of the ISLT timescale algorithm is to form a synthetic timescale with a more stable frequency by using the satellite clocks and ISL measurements. The computing framework of the KPW timescale was used for the ISLT timescale in this research. Furthermore, optimal weights that minimize the increment of the timescale are proposed and adjusted to the ensemble of clocks.

### 3.1. Basic ISLT Timescale Equation

Joint timekeeping means the clock ensemble maintains and is synchronized to a uniform time reference, which is essentially the weighted average of the ensemble of timekeeping clocks:

(4) $$\mathrm{ISLT}\left(t\right)=\sum_{i=1}^{n}w_{i}h_{i}\left(t\right)$$

where $h_{i}\left(t\right)$ is the clock reading of satellite *i*, and $w_{i}$ is, accordingly, the weight that satisfies the constraint:

(5) $$\sum_{i=1}^{n}w_{i}\left(t\right)=1$$

A brief explanation of Equation (4) is that the ISLT is the weighted average of readings of the clock ensemble. Though the clock reading is unmeasurable, the ISLT can be defined by its deviation relative to the GNSS system time, and Equation (4) is reformulated as:

(6) $$x_{e}\left(t\right)=\sum_{i=1}^{n}w_{i}\left(t\right)x_{i}\left(t\right)$$

where $x_{i}\left(t\right)$ and $x_{e}\left(t\right)$ are the absolute time deviation of satellite *i* and ISLT, respectively.

The essential purpose of forming ISLT is to improve frequency stability. To avoid the time or frequency steps because of the variation of the number of clock ensemble, the time deviation of each timekeeping clock should be predicted, and the time deviation measurement value can be detrended with the predicted deviation:

(7) $$x_{e}\left(t\right)=\sum_{i=1}^{n}w_{i}\left(t\right)\left[x_{i}\left(t\right)-{\hat{x}}_{ie}\left(t\right)\right]$$

where ${\hat{x}}_{ie}\left(t\right)$ is the predicted time deviation of satellite *i* relative to the ISLT. Equation (7) is the so-called basic timescale equation of the ISLT. When operating autonomously, the navigation satellite cannot establish a time comparison link with the ground station. Therefore, the absolute time deviation *x_i_* is also unmeasurable. Fortunately, the time deviations of slave satellites relative to the master satellite can be estimated by two-way inter-satellite ranging. Another form of the basic ISLT timescale equation is:

(8) $$x_{e1}\left(t\right)=\sum_{i=1}^{n}w_{i}\left(t\right)\left[x_{i1}\left(t\right)-{\hat{x}}_{ie}\left(t\right)\right]$$

where $x_{e1}\left(t\right)=x_{e}\left(t\right)-x_{1}\left(t\right)$ is the time deviation of the ISLT relative to the reference clock and $x_{i1}\left(t\right)$ is the time deviation of satellite *i* relative to the reference clock, as obtained from an inter-satellite time comparison. The time deviation of the ISLT relative to any satellite *i* is calculated by the relationship:

(9) $$x_{ei}\left(t\right)=x_{e1}\left(t\right)-x_{i1}\left(t\right)$$

According to the basic timescale Equation (8), three elements—inter-satellite time comparison, the short-term prediction of $x_{ie}\left(t\right)$ in the calculation cycle, the weight adjustment—are crucial for the ISLT.

### 3.2. Inter-Satellite Time Comparison

BeiDou inter-satellite links employ a system of time division, based on which the dual one-way ranging of a satellite pair is completed in two contiguous time intervals. Satellite B is assumed to send out a ranging signal at epoch *t*_s1_. Satellite A receives the signal at *t*_r1_ and sends out another ranging signal at *t*_s2_ (*t*_s2_ > *t*_r1_) that is received by satellite B at *t*_r2_. The sending moments *t*_s2_ and *t*_s1_ and the receiving moments *t*_r1_ and *t*_r2_ are the local time of satellites A and B, respectively, whose deviations from the system time are clock deviations $\delta t_{A}$ and $\delta t_{B}$. The relative clock deviation from inter-satellite time comparison is as follows:

(10) $$\delta t_{\mathrm{AB}}=\delta t_{A}-\delta t_{B}$$

Based on the process of non-simultaneous bidirectional ranging, a pair of ranging equations of two linking satellites are established:

(11) $$\left\{ \begin{array}{l} \rho_{\mathrm{AB}}=\left|R_{A}\left(t_{r1}\right)-R_{B}\left(t_{s1}\right)\right|+c\left[\delta t_{A}\left(t_{r1}\right)-\delta t_{B}\left(t_{s1}\right)\right]+c\tau_{A}^{r}+c\tau_{B}^{s}+\Delta \rho_{\mathrm{AB}}^{\mathrm{sys}}+\varepsilon_{\mathrm{AB}}\\ \rho_{\mathrm{BA}}=\left|R_{B}\left(t_{r2}\right)-R_{A}\left(t_{s2}\right)\right|+c\left[\delta t_{B}\left(t_{r2}\right)-\delta t_{A}\left(t_{s2}\right)\right]+c\tau_{B}^{r}+c\tau_{A}^{s}+\Delta \rho_{\mathrm{BA}}^{\mathrm{sys}}+\varepsilon_{\mathrm{BA}} \end{array}\right.$$

where $\Delta \rho_{\mathrm{AB}}^{\mathrm{sys}}$ and $\Delta \rho_{\mathrm{BA}}^{\mathrm{sys}}$ are systematic errors, e.g., the phase center offset of the ISL antennas and the relativistic effects, which can be accurately modeled. $\varepsilon_{\mathrm{AB}}$ and $\varepsilon_{\mathrm{BA}}$ are the ranging noise. Sending delays $\tau_{A}^{s}$ and $\tau_{B}^{s}$ and receiving delays $\tau_{A}^{r}$ and $\tau_{B}^{r}$ can be calibrated onboard with a self-closed loop of transmitting and receiving [19].

Correcting the systematic errors and channel delays, the original ranging equations are reformed as:

(12) $$\left\{ \begin{array}{l} {\bar{\rho }}_{\mathrm{AB}}=\left|R_{B}\left(t_{r1}\right)-R_{A}\left(t_{s1}\right)\right|+c\delta t_{B}\left(t_{r1}\right)-c\delta t_{A}\left(t_{s1}\right)+{\bar{\varepsilon }}_{\mathrm{AB}}\\ {\bar{\rho }}_{\mathrm{BA}}=\left|R_{A}\left(t_{r2}\right)-R_{B}\left(t_{s2}\right)\right|+c\delta t_{A}\left(t_{r2}\right)-c\delta t_{B}\left(t_{s2}\right)+{\bar{\varepsilon }}_{\mathrm{BA}} \end{array}\right.$$

where ${\bar{\rho }}_{\mathrm{AB}}$ and ${\bar{\rho }}_{\mathrm{BA}}$ are the corrected pseudoranges. The dual one-way pseudoranges need to be reduced to the reference epoch *t*_0_, given as *t*_0_ = (*t*_r1_ + *t*_r2_)/2, and the original observations are demonstrated as:

(13) $$\left\{ \begin{array}{l} {\bar{\rho }}_{\mathrm{AB}}=\left|R_{A}\left(t_{0}\right)-R_{B}\left(t_{0}\right)\right|+c\left[\delta t_{A}\left(t_{0}\right)-\delta t_{B}\left(t_{0}\right)\right]+\Delta \rho_{\mathrm{AB}}+{\bar{\varepsilon }}_{\mathrm{AB}}\\ {\bar{\rho }}_{\mathrm{BA}}=\left|R_{B}\left(t_{0}\right)-R_{A}\left(t_{0}\right)\right|+c\left[\delta t_{B}\left(t_{0}\right)-\delta t_{A}\left(t_{0}\right)\right]+\Delta \rho_{\mathrm{BA}}+{\bar{\varepsilon }}_{\mathrm{BA}} \end{array}\right.$$

where $\Delta \rho_{\mathrm{AB}}$ and $\Delta \rho_{\mathrm{BA}}$ are the pseudorange corrections from observation epoch to reduction epoch *t*_0_, and correction values are calculated with predicted orbit and clock deviation parameters:

(14) $$\left\{ \begin{array}{l} \Delta \rho_{\mathrm{AB}}=\left|{\tilde{R}}_{A}\left(t_{r1}\right)-{\tilde{R}}_{B}\left(t_{s1}\right)\right|-\left|{\tilde{R}}_{A}\left(t_{0}\right)-{\tilde{R}}_{B}\left(t_{0}\right)\right|+c\left\{\left[\delta {\tilde{t}}_{A}\left(t_{r1}\right)-\delta {\tilde{t}}_{B}\left(t_{s1}\right)\right]-\left[\delta {\tilde{t}}_{A}\left(t_{0}\right)-\delta {\tilde{t}}_{B}\left(t_{0}\right)\right]\right\}\\ \Delta \rho_{\mathrm{BA}}=\left|{\tilde{R}}_{B}\left(t_{r2}\right)-{\tilde{R}}_{A}\left(t_{s2}\right)\right|-\left|{\tilde{R}}_{A}\left(t_{0}\right)-{\tilde{R}}_{B}\left(t_{0}\right)\right|+c\left\{\left[\delta {\tilde{t}}_{B}\left(t_{r2}\right)-\delta {\tilde{t}}_{A}\left(t_{s2}\right)\right]-\left[\delta {\tilde{t}}_{B}\left(t_{0}\right)-\delta {\tilde{t}}_{A}\left(t_{0}\right)\right]\right\} \end{array}\right.$$

The superscript “~” stands for predicted values. By correcting the original pseudoranges with items above, we can get the reduction pseudoranges:

(15) $$\left\{ \begin{array}{l} {\tilde{\rho }}_{\mathrm{AB}}=\left|R_{A}\left(t_{0}\right)-R_{B}\left(t_{0}\right)\right|+c\left[\delta t_{A}\left(t_{0}\right)-\delta t_{B}\left(t_{0}\right)\right]+{\tilde{\varepsilon }}_{\mathrm{AB}}\\ {\tilde{\rho }}_{\mathrm{BA}}=\left|R_{B}\left(t_{0}\right)-R_{A}\left(t_{0}\right)\right|+c\left[\delta t_{B}\left(t_{0}\right)-\delta t_{A}\left(t_{0}\right)\right]+{\tilde{\varepsilon }}_{\mathrm{BA}} \end{array}\right.$$

The difference of the two reduction pseudoranges is free of the orbits and could be used to determine the inter-satellite clock deviation at *t*_0_:

(16) $$\delta t_{\mathrm{AB}}\left(t_{0}\right)=\delta t_{A}\left(t_{0}\right)-\delta t_{B}\left(t_{0}\right)\;=\frac{{\tilde{\rho }}_{\mathrm{AB}}-{\tilde{\rho }}_{\mathrm{BA}}}{2c}+\varepsilon$$

where ε is the equivalent error of inter-satellite time comparison.

### 3.3. Short-Term Prediction of Clock Deviation

Both the deterministic and stochastic components are modeled with the state equations of atomic clocks in a Kalman filter. The time deviation, frequency deviation, and frequency drift can be estimated synchronously and used for short-term prediction. In the Kalman filter benchmark, the state space of clocks should be built first. Considering the three clock states above, a three-stage stochastic differential model is:

(17) $$\left\{ \begin{array}{l} dx\left(t\right)=y\left(t\right)\cdot dt+\sigma_{1}\cdot dW_{1}\left(t\right)\\ dy\left(t\right)=z\left(t\right)\cdot dt+\sigma_{2}\cdot dW_{2}\left(t\right)\\ dz\left(t\right)=\sigma_{3}\cdot dW_{3}\left(t\right) \end{array}\right.$$

where *x*(*t*) is the time deviation of a physical clock relative to an ideal timescale, which is a realization of Systeme International d’Unites (SI) second, *y*(*t*) is a component of the frequency deviation with a random walk, and *z*(*t*) is a component of the frequency drift with random walk. $W_{1}\left(t\right)$, $W_{2}\left(t\right)$ and $W_{3}\left(t\right)$ are three independent Wiener processes, which are known as the random walk noise, whose derivatives are white noise, and whose integrals are random run noise [20]. Accordingly, their diffusion coefficients are $\sigma_{1}$, $\sigma_{2}$ and $\sigma_{3}$, respectively, representing the noise intensities of white frequency noise (WFM), random walk frequency noise (RWFM), and random run frequency noise (RRFM).

Given initial conditions at the moment $t_{0}$

(18) $$\left\{ \begin{array}{l} x\left(t_{0}\right)=x_{0}\\ y\left(t_{0}\right)=y_{0}\\ z\left(t_{0}\right)=z_{0} \end{array}\right.,$$

a closed-form solution of Equation (10) is reached:

(19) $$\left\{ \begin{array}{l} x\left(t\right)=x_{0}+y_{0}t+1/2\cdot z_{0}\cdot t^{2}+\sigma_{1}\cdot W_{1}\left(t\right)+\sigma_{2}\cdot \int_{0}^{t}W_{2}\left(s\right)ds+\sigma_{3}\cdot \int_{0}^{t}\int_{0}^{u}W_{3}\left(s\right)dsdu\\ y\left(t\right)=y_{0}+z_{0}t+\sigma_{2}\cdot W_{2}\left(t\right)+\sigma_{3}\cdot \int_{0}^{t}W_{3}\left(s\right)ds\\ z\left(t\right)=z_{0}+\sigma_{3}\cdot W_{3}\left(t\right) \end{array}\right.$$

Note the sampling interval as τ, and the discrete form of Equation (12) is:

(20) $$\left\{ \begin{array}{l} x\left(t\right)=x\left(t-\tau \right)+y\left(t-\tau \right)\cdot \tau +1/2\cdot z\left(t-\tau \right)\cdot \tau^{2}+\gamma_{x}\left(t\right)\\ y\left(t\right)=y\left(t-\tau \right)+z\left(t-\tau \right)\cdot \tau +\gamma_{y}\left(t\right)\\ z\left(t\right)=z\left(t-\tau \right)+\gamma_{z}\left(t\right) \end{array}\right.$$

The noise vector $\gamma \left(t\right)={\left[\begin{array}{ccc} \gamma_{x}\left(t\right) & \gamma_{y}\left(t\right) & \gamma_{z}\left(t\right) \end{array}\right]}^{T}$ obeys a bivariate Gaussian distribution with the mean equal to zero, and the covariance matrix as follows:

(21) $$q=\left[\begin{array}{ccc} \sigma_{1}^{2}\tau +\frac{\sigma_{2}^{2}\tau^{3}}{3}+\frac{\sigma_{3}^{2}\tau^{5}}{20} & \frac{\sigma_{2}^{2}\tau^{2}}{2}+\frac{\sigma_{3}^{2}\tau^{4}}{8} & \frac{\sigma_{3}^{2}\tau^{3}}{6}\\ \frac{\sigma_{2}^{2}\tau^{2}}{2}+\frac{\sigma_{3}^{2}\tau^{4}}{8} & \sigma_{2}^{2}\tau +\frac{\sigma_{3}^{2}\tau^{3}}{3} & \frac{\sigma_{3}^{2}\tau^{2}}{2}\\ \frac{\sigma_{3}^{2}\tau^{3}}{6} & \frac{\sigma_{3}^{2}\tau^{2}}{2} & \sigma_{3}^{2}\tau \end{array}\right]$$

The state vector of the Kalman filter consists of the states of *n* atomic clocks:

(22) $$X={\left[\begin{array}{ccccccc} x_{1} & y_{1} & z_{1} & \ldots & x_{n} & y_{n} & z_{n} \end{array}\right]}^{T}$$

Let $X_{k}=X\left(t_{k}\right)$, a state equation of the clock ensemble, be written as:

(23) $$X_{k}=\Phi_{k}X_{k-1}+{ϒ}_{k}$$

The state transition matrix is

(24) $$\Phi_{k}=\left[\begin{array}{ccc} \phi_{1,k} & & \\ & \ddots & \\ & & \phi_{n,k} \end{array}\right]$$

whose diagonal element $\phi_{i,k}$ is the state transition matrix of clock *i* with:

(25) $$\phi_{i,k}=\left[\begin{array}{ccc} 1 & \tau & \tau^{2}/2\\ 0 & 1 & \tau \\ 0 & 0 & 1 \end{array}\right],$$

where the sampling time is $\tau =t_{k}-t_{k-1}$. The noise vector is:

(26) $$ϒ={\left[\begin{array}{ccccccc} \gamma_{1,x} & \gamma_{1,y} & \gamma_{1,z} & \ldots & \gamma_{n,x} & \gamma_{n,y} & \gamma_{n,z} \end{array}\right]}^{T}$$

whose covariance matrix is:

(27) $$Q=\left[\begin{array}{ccc} q_{1} & & \\ & \ddots & \\ & & q_{n} \end{array}\right]$$

where $q_{i}$ is the covariance matrix of clock *i*, defined by Equation (14).

In each calculation cycle of the ISLT, all the salve satellites compare their clock with the reference clock. The time measurement equation is formed as:

(28) $$Z\left(t_{k}\right)=HX\left(t_{k}\right)+V\left(t_{k}\right)$$

where $V\left(t_{k}\right)$ is the noise of comparison links whose covariance matrix is *R*. The relative deviation vector is $Z={\left[\begin{array}{cccc} x_{21} & x_{31} & \ldots & x_{n1} \end{array}\right]}^{T}$, and the measurement matrix is:

(29) $$H=\left[\begin{array}{cccccccccc} -1 & 0 & 0 & 1 & 0 & 0 & \cdots & 0 & 0 & 0\\ & & & & & & \vdots & & & \\ -1 & 0 & 0 & 0 & 0 & 0 & \cdots & 1 & 0 & 0 \end{array}\right]$$

The estimation of 3*n* states based on *n* − 1 measurements with a Kalman filter follows the steps:

(30) $$\left\{ \begin{array}{l} {\hat{X}}_{k,k-1}=\Phi_{k}{\hat{X}}_{k-1}\\ P_{k,k-1}=\Phi_{k}P_{k-1}\Phi_{k}^{T}+Q\\ K_{k}=P_{k,k-1}H^{T}{\left(HP_{k,k-1}H^{T}+R\right)}^{-1}\\ {\hat{X}}_{k}={\hat{X}}_{k,k-1}+K_{k}\left(Z_{k}-H{\hat{X}}_{k,k-1}\right)\\ P_{k}=\left(I-K_{k}H\right)P_{k,k-1} \end{array}\right.$$

where $P=E\left[\left(X-\hat{X}\right){\left(X-\hat{X}\right)}^{T}\right]$ is the covariance matrix of estimation errors.

Because of the unobservability of the inter-satellite comparison network, estimation errors of the Kalman filter keep divergent when estimating 3*n* states with *n* − 1 relative measurements. However, the errors of frequency deviations and drifts diverge observably more slowly than time deviations, and they can be used to reconstitute the time deviation [14]:

(31) $${\hat{x}}_{ie}\left(t\right)=x_{ie}\left(t-\tau \right)+\tau \cdot {\hat{y}}_{i,Kalman}\left(t-\tau \right)+\frac{1}{2}\cdot \tau^{2}\cdot {\hat{z}}_{i,Kalman}\left(t-\tau \right),$$

which is used as the predicted deviation in the basic timescale equation.

### 3.4. Optimal Weights

According to the bivariate state equation, the frequency deviation ${\hat{y}}_{i,Kalman}$ and drift ${\hat{z}}_{i,Kalman}$, as estimated by the Kalman filter, are only influenced by RWFM and RRFM, respectively. Therefore, the reconstituted time deviation ${\hat{x}}_{ie}\left(t\right)$ is not influenced by WFM. The weights employed in [14] were based on the approximation that $x_{i}\left(t\right)-{\hat{x}}_{ie}\left(t\right)$ only includes WFM, and they were adjusted according to the intensity of WFM. In this research, the optimal weights were adjusted to minimize the increment of the synthetic timescale.

The basic ISLT timescale equation can be translated by using the reconstituted time deviation as predicted values into:

(32) $$x_{e}\left(t\right)=\sum_{i=1}^{n}w_{i}\left(t\right)\left[x_{i}\left(t\right)-x_{ie}\left(t-\tau \right)-\tau \cdot {\hat{y}}_{i,Kalman}\left(t-\tau \right)-\frac{1}{2}\cdot \tau^{2}\cdot {\hat{z}}_{i,Kalman}\left(t-\tau \right)\right]$$

Based on the definition of relative time deviation, Equation (32) is rewritten as:

(33) $$x_{e}\left(t\right)=x_{e}\left(t-\tau \right)+\sum_{i=1}^{n}w_{i}\left(t\right)\left[x_{i}\left(t\right)-x_{i}\left(t-\tau \right)-\tau \cdot {\hat{y}}_{i,Kalman}\left(t-\tau \right)-\tau^{2}/2\cdot {\hat{z}}_{i,Kalman}\left(t-\tau \right)\right]$$

Define the increment of a timescale as:

(34) $$\Delta_{\tau }x\left(t\right)=x\left(t\right)-x\left(t-\tau \right)$$

Combined with the second and third relationships in Equation (20), the increment form of the basic timescale equation is obtained:

(35) $$\Delta_{\tau }x_{e}\left(t\right)=\sum_{i=1}^{n}w_{i}\left(t\right)\left\{\tau \left[y_{i}\left(t-\tau \right)-{\hat{y}}_{i,Kalman}\left(t-\tau \right)\right]+\tau^{2}/2\left[z_{i}\left(t-\tau \right)-{\hat{z}}_{i,Kalman}\left(t-\tau \right)\right]+\gamma_{i,x}\left(t\right)\right\}$$

The weight vector is:

(36) $$W=\left[\begin{array}{cccc} w_{1}\left(t\right) & w_{2}\left(t\right) & \ldots & w_{n}\left(t\right) \end{array}\right]$$

A column vector is written as:

(37) $$M=\left[\begin{array}{c} \tau \cdot \left[y_{1}\left(t-\tau \right)-{\hat{y}}_{1,Kalman}\left(t-\tau \right)\right]+\tau^{2}/2\left[z_{1}\left(t-\tau \right)-{\hat{z}}_{1,Kalman}\left(t-\tau \right)\right]+\gamma_{1,x}\left(t\right)\\ \tau \cdot \left[y_{2}\left(t-\tau \right)-{\hat{y}}_{2,Kalman}\left(t-\tau \right)\right]+\tau^{2}/2\left[z_{2}\left(t-\tau \right)-{\hat{z}}_{2,Kalman}\left(t-\tau \right)\right]+\gamma_{2,x}\left(t\right)\\ \vdots \\ \tau \cdot \left[y_{n}\left(t-\tau \right)-{\hat{y}}_{n,Kalman}\left(t-\tau \right)\right]+\tau^{2}/2\left[z_{n}\left(t-\tau \right)-{\hat{z}}_{n,Kalman}\left(t-\tau \right)\right]+\gamma_{n,x}\left(t\right) \end{array}\right]$$

Equation (35) is reformed as:

(38) $$\Delta_{\tau }x_{e}\left(t\right)=WM$$

The variance of the timescale increment is accordingly:

(39) $$Var\left[\Delta_{\tau }x_{e}\left(t\right)\right]=WFW^{T}$$

*F* is the covariance matrix of *M*, satisfying:

(40) $$F_{ij}=\tau^{2}P\left(y_{i},y_{j}\right)+\frac{\tau^{3}}{2}P\left(y_{i},z_{j}\right)+\frac{\tau^{3}}{2}P\left(z_{i},y_{j}\right)+\frac{\tau^{4}}{4}P\left(z_{i},z_{j}\right)+q_{i,11}\delta_{ij}$$

where *P* is the Kalman covariance of relevant states and $\delta_{ij}$ meets:

(41) $$\delta_{ij}=\{\begin{array}{c} 1,\;i=j\\ 0,\;i\neq j \end{array}$$

Weights are adjusted to minimize the increment of the timescale means to determine *W* to minimize Equation (39). With the constraint Equation (5), the objective function is:

(42) $$J=WFW^{T}+2\lambda \left(1-W\cdot 1_{n}\right)$$

where λ is a Lagrangian multiplier and $1_{n}$ is a column vector consisting of *n* ones. Our goal is translated to solve the equations:

(43) $$\left\{ \begin{array}{l} \frac{\partial J}{\partial W}=2FW^{T}-2\lambda \cdot 1_{n}=0\\ \frac{\partial J}{\partial \lambda }=2\lambda \left(1-W\cdot 1_{n}\right)=0 \end{array}\right.$$

That is:

(44) $$\left[\begin{array}{cccc} F_{11} & \cdots & F_{1n} & -1\\ \vdots & \vdots & \vdots & \vdots \\ F_{n1} & \cdots & F_{nn} & -1\\ 1 & \cdots & 1 & 0 \end{array}\right]\left[\begin{array}{c} w_{1}\left(t\right)\\ \vdots \\ w_{n}\left(t\right)\\ \lambda \end{array}\right]=\left[\begin{array}{c} 0\\ \vdots \\ 0\\ 1 \end{array}\right]$$

Let:

(45) $$A=\left[\begin{array}{cccc} F_{11} & \cdots & F_{1n} & -1\\ \vdots & \vdots & \vdots & \vdots \\ F_{n1} & \cdots & F_{nn} & -1\\ 1 & \cdots & 1 & 0 \end{array}\right]$$

when *A* is an invertible matrix, there is a unique solution of Equation (44). Let *C* = *A*^−1^, and the optimal weight of clock *i* is:

(46) $$w_{i}\left(t\right)=C_{i,n+1}$$

With this strategy of weighting, the weights of the ensemble of satellite clocks are not only determined by the intensity of WFM but also by the filtering covariance of frequency deviations and drifts.

## 4. Performance Evaluation of ISLT

Nine BeiDdou-3 medium earth orbit (MEO) satellites, which operated normally without phase or frequency modulation during the analysis period, were selected for joint timekeeping analysis. The nine satellites are equipped with five rubidium (Rb) clocks and four passive hydrogen masers (PHMs), as illustrated in Table 1. 

### 4.1. Clock Noise Identification

MPOD clock products were collected by the International GNSS Service (IGS) Multi-GNSS Experiment (MGEX) analysis centers (ACs) from 1 March to 31 March 2019—31 days in total for satellite clock identification.

The short-term prediction precision of the three-state Kalman filter relied on the modeling of WFM, RWFM and RRFM and the estimation of the corresponding diffusion coefficients based on the clock products. The clock deviation outliers were always submerged because of the large scale of clock deviations. Clock deviations were therefore transformed to frequency deviations through first-order difference for gross error detection:

(47) $$y_{k}=\frac{x_{k+1}-x_{k}}{\tau_{0}},\;k=1,\ldots ,N-1$$

where *N* is the amount of clock deviation data and $\tau_{0}$ is the sample interval of 30 s. The median method (MAD) is used to detect gross errors, and when the frequency deviation:

(48) $$\left|y_{k}\right|>m+p\cdot \mathrm{MAD},$$

$y_{k}$ is treated as a gross error and eliminated from the original data. $m=\mathrm{Median}\left(y_{k}\right)$, $\mathrm{MAD}=\mathrm{Median}\left(\left|y_{k}-m\right|/0.6745\right)$. Median is the operation of estimating the median. *p* was taken as 5 in this research. After removing the gross errors, the missing data were assigned by interpolation.

**Figure 2:**
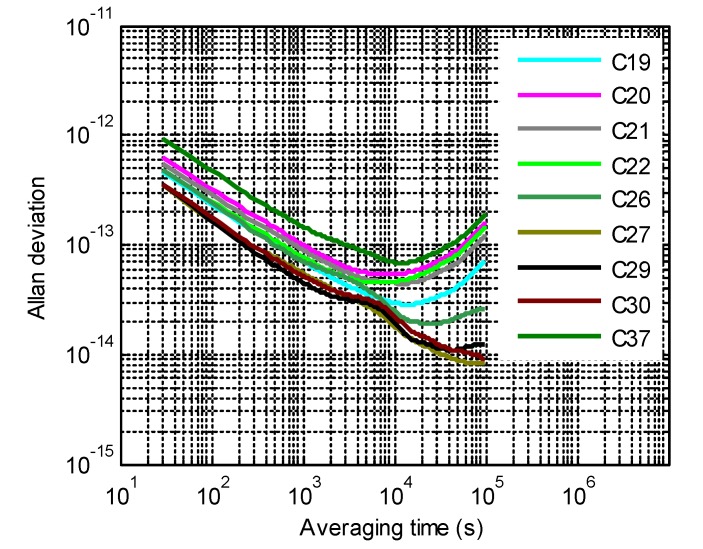
Allan deviations (ADEVs) of detrended clock deviations.

The MPOD clock products have absorbed the orbit mismodeling errors because of the correlation between the satellite orbits and clocks. In order to reliably identify the background noises of BeiDou spaceborne clocks, the periodic signal at 1, 2, 3, and 4 cycle(s) per revolution (cpr) is removed from clock deviations [16]. According to the detrended clock deviations, Allan deviations (ADEVs) were computed and are shown in Figure 2. The three noise components are related to Allan variances of the clocks by:

(49) $$\sigma_{y}^{2}\left(\tau \right)=\frac{\sigma_{1}^{2}}{\tau }+\frac{\sigma_{2}^{2}\tau }{3}+\frac{\sigma_{3}^{2}\tau^{3}}{20}$$

which means that the Allan deviation is a function of the averaging time that is formulated as $\sigma_{y}\left(\tau \right)=A\tau^{\mu }$ or the logarithm form $\mathrm{lg}\sigma_{y}\left(\tau \right)=\mathrm{lg}A+\mu \mathrm{lg}\tau$, μ is the curve slope of $\mathrm{lg}\sigma_{y}\left(\tau \right)$~lgτ, equaling −0.5, 0.5 and 1.5 for WFM, RWFM and RRFM, respectively, and the constant term lgA relates to the diffusion coefficients. If the curve $\mathrm{lg}\sigma_{y}\left(\tau \right)$~lgτ is fit piecewise with a linear model, the clock noises are identified as in Table 2.

In general, the onboard Rb clocks are mainly affected by white frequency noises and random walk noises, while the PHMs are only influenced by white frequency noises. Since no random run frequency noise was observed for all the nine clocks, with an averaging time of less than 100,000 s, the corresponding diffusion coefficients were taken as zeros and the same to the RWFM diffusion coefficients of PHMs.

### 4.2. Inter-Satellite Clock Deviations

**Figure 3:**
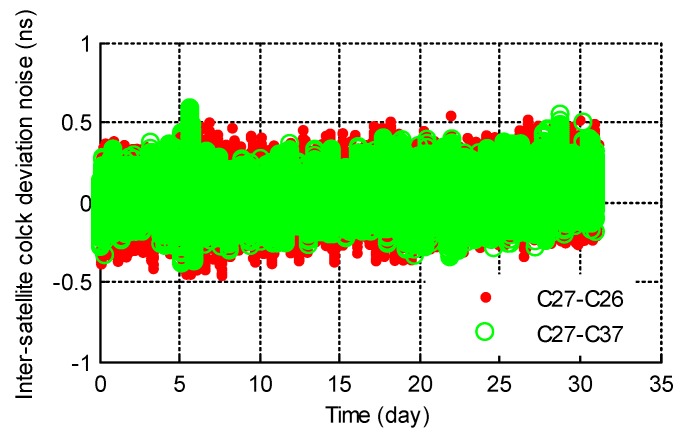
The precision of inter-satellite time comparison.

The inter-satellite time comparison performance was analyzed with real ISL measurements of the 31 days. ISL relative clock deviations were calculated with Equation (16). The daily inter-satellite clock deviations were fitted with two-order polynomials to remove the initial clock deviations, frequency deviations and frequency drifts. The detrended clock deviations of pairs C27−C26 and C27−C37 are illustrated in Figure 3 as examples, which demonstrate the comparison precision was better than 0.2 ns (root mean square error: RMSE).

**Figure 4:**
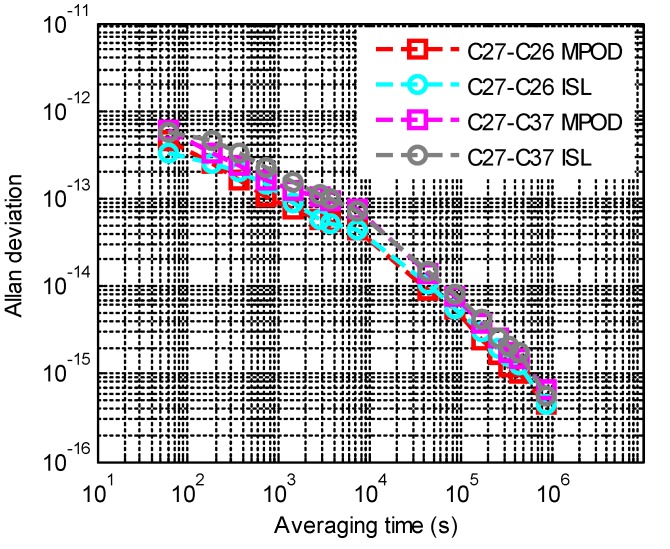
The noise character of relative clock deviations.

By making difference between the MPOD clock deviations of a satellite pair, we were also able to get the inter-satellite relative clock deviations. The deterministic components were removed from the MPOD relative clock deviations. The noise characters of ISL and MPOD relative clock deviations were analyzed with Allan deviations, as shown in Figure 4. It should be noted that the noise of ISL clock deviations was filtered through a low pass filter, and the cycles per revolution were removed from MPOD clock deviations. According to the result, the noise characters of the two types of relative clock deviations were consistent.

### 4.3. Frequency Stability Analysis

The establishment of the ISLT requires periodic time comparison between the master satellite and slave satellites, a process which is not satisfied by the actual states of BeiDou ISLs. As the noise characters of ISL and MPOD relative clock deviations are consistent, the difference values of MPOD clock deviations were used as inter-satellite clock deviations in the analysis. By implementing the ISLT timescale algorithm, we obtained the ISLT expressed as the clock deviation relative to the reference clock. By using Equation (1), the relative deviation was translated to the absolute clock deviation relative to the same reference timescale with the satellite clocks.

**Figure 5:**
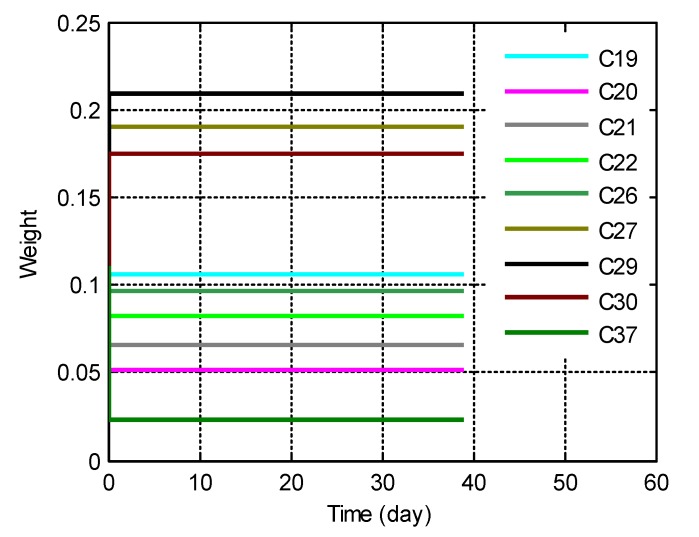
Weights of the clock ensemble (1 March–31 March 2019).

The weight allocation according to Equation (39) of the clock ensemble is shown in Figure 5. The frequency of C19 was more stable in the short term (averaging time smaller than 7000 s) than that of C26 and was allocated with a larger weight, while the frequency of C27 was more stable in the long term (averaging time larger than 7000 s) than that of C30 and was allocated with a larger weight. This contrast demonstrates that the weights of the ISLT timescale had comprehensively considered both the long-term and short-term frequency stability.

**Figure 6:**
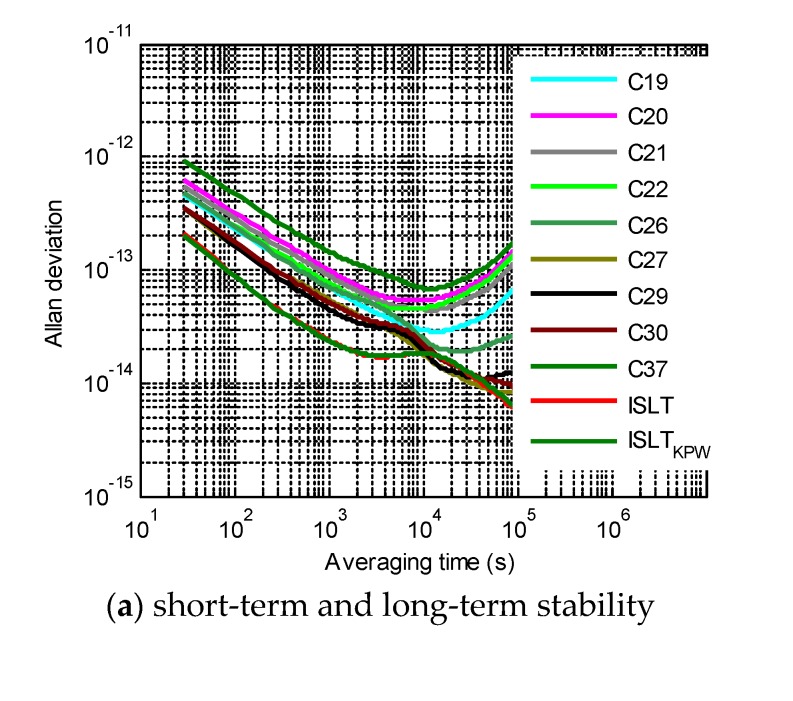

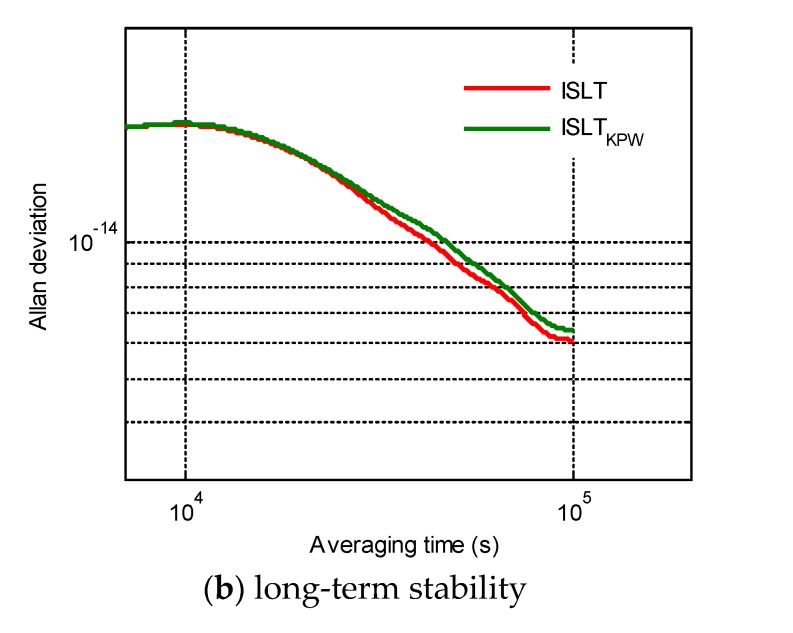
Frequency stability comparison of different timescales.

The Allan deviations of the satellite clocks are drawn again in Figure 6 and compared with the ISLT jointly kept by the nine satellite clocks. The ISLT_KPW_ was generated by the KPW algorithm in [14], while the ISLT was generated by the algorithm in this research. The short-term stability of the ISLT and ISLT_KPW_ was almost the same, but the long-term frequency of the ISLT was more stable, demonstrating that our weights were better (Figure 6b). Among the Rb clocks, the C19 clock was the most stable, and the C37 clock was the worst. The frequency stability after 10,000 s of averaging time of all the Rb clocks decreased due to the influence of RWFM. The frequencies of PHMs are generally more stable than Rb clocks. Expect for those of C26, the PHMs of other three satellites showed a similar frequency stability, with their daily stability reaching 8 × 10^−15^, so they were allocated with the three largest weights.

For an averaging time of less than 7000 s, the frequency of the ISLT was more stable than that of all the spaceborne clocks. However, the Allan deviation slightly increased around 10,000 s of averaging time, possibly because of the mismodeling non-power-law behaviors of PHMs or the flicker frequency noises (FFM) of Rb clocks. Even so, the daily stability of the ISLT reached 6 × 10^−15^, which was better than the best PHM of the four satellites.

**Figure 7:**
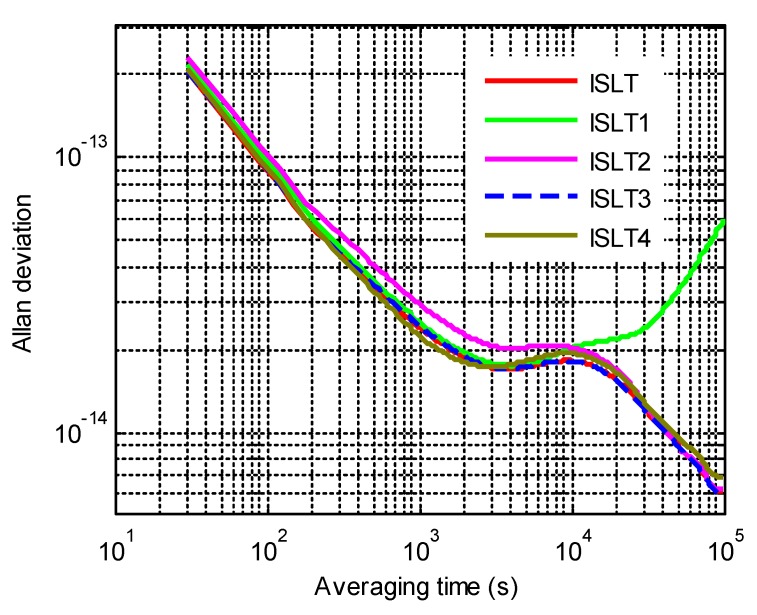
The frequency stability of the ISLTs kept by different clock ensembles.

Figure 7 demonstrates the ISLTs kept by different ensembles of satellite clocks, as illustrated in Table 3 to evaluate the influence of the clock ensemble. Since the long-term stability of PHMs was obviously superior to Rb clocks, the frequency of the ISLT2 was more stable than the ISLT1 in the long term and approaches to the ISLT after 30,000 s of averaging time. Meanwhile, the short-term stability of the ISLT1 was better than the ISLT2 because the weights of the ISLT1 were more dependent on the short-term stability of the Rb clock ensemble. The ADEVs of the ISLT3 and ISLT4 indicate that the malfunction of a timekeeping clock has little influence on the stability of the ISLT, especially for the one with a small weight.

### 4.4. Predictability Improvement

The prediction accuracy of satellite clocks is important for positioning and timing performance. In this part, we evaluate the predictability of the ISLT. For the short-term prediction, a linear model was used to fit the prediction parameters by using the MPOD clock deviations that were collected every two hours and then used to evaluate the predictability over the next two hours. The prediction accuracy was assessed with the root-mean-square error (RMSE) and is displayed Table 4. The optimal short-term prediction accuracy of the PHM was 0.27 ns, as in [3]. Except for C19 and C26, the short-term prediction accuracy of PHMs was generally superior to the Rb clocks. The prediction accuracy of the ISLT was 0.18 ns better than all the timekeeping clocks.

For the long-term prediction, a linear model was used to fit the prediction parameters by using the MPOD clock deviations that were collected every 24 h and then used to evaluate the predictability over the next 10 h. The prediction accuracy is displayed in Table 5. The optimal long-term prediction accuracy of the PHM was 1.16 ns worse than the result in [3]. The reason for this is that the MPOD clock deviation absorbed the orbit error, while the TWSTFT clock deviation did not. Except for C26, the long-term prediction accuracy of PHMs was generally superior than all the Rb clocks. The prediction accuracy of the ISLT was 1.05 ns better than all the timekeeping clocks.

## 5. Summary and Conclusions

We proposed a joint timekeeping method with ISLs to improve the timekeeping of a navigation satellite constellation. Different from the distributed mode, the navigation satellites jointly established and maintained a uniform time reference through a timekeeping framework with an ISLT. A weighted average timescale algorithm that minimized the increment of the timescale was proposed and used to establish the ISLT and to improve frequency stability and predictability. Data from nine BeiDou-3 MEO satellites that were equipped with five Rb clocks and four PHMs were used for performance evaluation.

The onboard Rb clocks are mainly affected by white frequency noises and random walk noises, and the PHMs are only influenced by white frequency noises with an averaging time of less than 100,000 s. The performances of PHMs are generally superior to Rb clocks. The daily stability of the clock ensemble was found to reach 8 × 10^−15^, the optimal short-term prediction accuracy was found to achieve 0.27 ns, and the long-term prediction accuracy was found to be 1.16 ns.

Based on the noise identification and inter-satellite clock deviations, the ISLT was calculated with the ISLT timescale algorithm. The short-term frequency of the ISLT was more stable than that of all the nine clocks, and its daily stability reached 6 × 10^−15^. The short-term and long-term prediction accuracy of the ISLT was 0.18 ns and 1.05 ns, respectively, which was better than all the timekeeping clocks. Furthermore, the ISLT was robust to the malfunction of an individual timekeeping clock, especially for one with a small weight.

## Figures and Tables

**Table 1:** Atomic clocks of nine BeiDou-3 satellites.

Serial Number	Satellite PRN	Nominal Clock
1	C19	Rb
2	C20	Rb
3	C21	Rb
4	C22	Rb
5	C26	PHM
6	C27	PHM
7	C29	PHM
8	C30	PHM
9	C37	Rb

**Table 2:** Noise diffusion coefficients of three BeiDou-3 satellites (passive hydrogen maser (PHM) indicated in italics).

PRN	Noise Diffusion Coefficients		
	$\sigma_{1}\;(s^{1/2})$	$\sigma_{2}\;(s^{-1/2})$	$\sigma_{3}\;(s^{-3/2})$
C19	2.38 × 10^−12^	5.66 × 10^−16^	/ ^1^
C20	3.42 × 10^−12^	1.82 × 10^−15^	/
C21	3.02 ×10^−12^	1.41 × 10^−15^	/
C22	2.68 ×10^−12^	1.67 × 10^−15^	/
*C26*	2.50 × 10^−12^	*/*	*/*
*C27*	1.78 × 10^−12^	*/*	*/*
*C29*	1.70 × 10^−12^	*/*	*/*
*C30*	1.86 × 10^−12^	*/*	*/*
C37	5.05 × 10^−12^	2.05 × 10^−15^	/

^1^ “/” means the noise is extremely small and not observed.

**Table 3:** The ensemble of satellite clocks that kept the ISLT.

ISLT Symbol	Clock Ensemble
ISLT	All the nine satellite clocks
ISLT1	All the five Rb clocks
ISLT2	All the four PHMs
ISLT3	C19 C20 C21 C22 C26 C27 C29 C30
ISLT4	C19 C20 C21 C22 C26 C29 C30 C37

**Table 4:** Short-term clock prediction accuracy.

PRN	Predicting RMS (ns)	PRN	Predicting RMS (ns)
C19	0.25	*C27*	*0.27*
C20	0.37	*C29*	*0.33*
C21	0.36	*C30*	*0.35*
C22	0.40	C37	0.80
*C26*	*0.46*	ISLT	0.18

**Table 5:** Long-term clock prediction accuracy.

PRN	Predicting RMS (ns)	PRN	Predicting RMS (ns)
C19	1.45	*C27*	*1.16*
C20	2.73	*C29*	*1.29*
C21	2.78	*C30*	*1.30*
C22	2.61	C37	4.67
*C26*	*2.28*	ISLT	1.05
